# New opportunities for the integration of microorganisms into biological pest control systems in greenhouse crops

**DOI:** 10.1007/s10340-016-0751-x

**Published:** 2016-03-21

**Authors:** Francisco Gonzalez, Cezary Tkaczuk, Mihaela Monica Dinu, Żaneta Fiedler, Stefan Vidal, Einat Zchori-Fein, Gerben J. Messelink

**Affiliations:** Department of Plant Protection Biology, Swedish University of Agricultural Sciences, Växtskyddsvägen 3, P.O. Box 23053, Alnarp, Sweden; Department of Plant Protection and Breeding, Siedlce University of Natural Sciences and Humanities, Prusa 14 Street, 08-110 Siedlce, Poland; Research-Development Institute for Plant Protection, Bd. Ion Ionescu de la Brad nr. 8, Sector 1, P.O. Box 013813, Bucharest, Romania; Department of Biological Control, Institute of Plant Protection – NRI, Władysława Węgorka 20 Street, 60-318 Poznań, Poland; Department of Crop Sciences, Agricultural Entomology, Georg-August-University, Grisebachstrasse 6, 37077 Goettingen, Germany; Department of Entomology, NeweYa’ar Research Center, P.O. Box 1021, 30095 Ramat Yishay, Israel; Wageningen UR Greenhouse Horticulture, PO Box 20, 2265 ZG Bleiswijk, The Netherlands

**Keywords:** Arthropod natural enemies, Microbials, Entomopathogens, Endophytes, Symbionts

## Abstract

Biological pest control with mass-produced arthropod natural enemies is well developed in greenhouse crops and has often resulted in the evolution of complex ecosystems with persistent populations of multiple arthropod natural enemy species. However, there are cases where arthropod natural enemies are either not effective enough, not available, or their use is rather costly. For these reasons, biological control based on microorganisms, also referred to as ‘microbials’, represents a complementary strategy for further development. Although commercially available microbials have been around for quite some time, research on and the applied use of combinations of arthropod natural enemies and microbials have remained relatively under explored. Here, we review current uses of entomopathogenic fungi, bacteria and viruses, and their possible direct and indirect effects on arthropod natural enemies in European greenhouses. We discuss how microbials might be combined with arthropod natural enemies in the light of new methodologies and technologies such as conservation biological control, greenhouse climate management, and formulation and delivery. Furthermore, we explore the possibilities of using other microorganisms for biological control, such as endophytes, and the need to understand the effect of insect-associated microorganisms, or symbionts, on the success of biological control. Finally, we suggest future research directions to optimize the combined use of microbials and arthropod natural enemies in greenhouse production.

## Key message

- The application of microbials for pest control in greenhouse crops should be integrated with the use of arthropod natural enemies. Here we review the current uses of entomopathogenic fungi, bacteria and viruses, and their possible direct and indirect effects on arthropod natural enemies.
- New approaches in the use of conservation biological control, greenhouse climate management, formulation, delivery and endophytic microorganisms could increase the various ways in which microbials can interact with arthropod natural enemies, and these interactions can be both positive and negative for pest control.
- A better understanding of these interactions offers new opportunities to optimize and further develop biological pest control.

## Introduction

Biological control of arthropod pests by arthropod natural enemies has been used successfully in greenhouse crops for decades (Pilkington et al. 2010). The protected environment of high-value greenhouse crops is particularly suitable for the effective functioning of commercially produced natural enemies and, globally, the majority of arthropod natural enemy species sold are used for augmentation in greenhouse crops (van Lenteren 2012). However, despite this success, there are still many cases where arthropod natural enemies are not used due to high costs or low efficacy.

Biological control agents based on entomopathogenic microorganisms (viruses, bacteria, fungi, etc.), also referred to as microbials, are often promoted as an alternative or back-up treatment when arthropod natural enemies are unavailable or not sufficiently effective (Chandler et al. 2011). The application of entomopathogens must be compatible with the arthropod natural enemies that are used in the same biological control system, and side effects of entomopathogens have therefore been studied extensively (Roy and Pell 2000). However, recent studies are increasingly exploring the wider properties of microorganisms, which suggest new opportunities for their use in biological control systems (Lacey et al. 2015). For example, several entomopathogenic fungi can also colonize plant tissues as endophytes and affect pests systemically via the plant (Vega et al. 2009). Furthermore, microorganisms that are symbionts of pests can influence the successes of both arthropod natural enemies and entomopathogens and therefore biological control (Zindel et al. 2011).

In this review, we discuss the need to use microbials and endophytes for pest control on the most important greenhouse crops. Greenhouse crops offer unique opportunities to design and optimize ecosystems through releases of arthropod natural enemies and by manipulating the greenhouse climate. We believe it is important to consider how new applications of microbials fit into such a designed ecosystem. We particularly address the means by which microbials and endophytes can be used to support and enhance biological control by arthropod natural enemies in different greenhouse cropping systems and discuss new developments, knowledge gaps and future prospects for the use of microbials in greenhouse crops. When discussing registered microbial products, we have focussed on entomopathogenic baculoviruses, bacteria and fungi that are currently registered for use in Europe. These groups are often considered as examples of biopesticides (Glare et al. 2012), alongside natural compounds and minerals. Here we will only consider living microorganisms, as this category of biopesticides requires application approaches that differ substantially from chemical pesticides and other nonliving biopesticides. Entomopathogenic nematodes are often also considered as microbials by the biocontrol industry, but despite their obvious importance, they will also be excluded from this review as, strictly speaking, they are not microorganisms but animals that use associated bacteria to kill their hosts (Kaya and Gaugler 1993). The majority of commercially available microbial products are based on the species *Bacillus thuringiensis* Berliner (Bt), but as this species has already been extensively reviewed (Sanahuja et al. 2011; Vachon et al. 2012), it will not be discussed in great detail here.

## Current status of microbials used for pest control in greenhouse crops

In Europe in 2010, the estimated sales of microbials based on entomopathogens, such as bacteria, viruses and fungi, was 42 million Euro, of which the majority (58 %) could be assigned to *B. thuringiensis* (Glare et al. 2012). Although the market for microbials is expected to increase substantially (Glare et al. 2012), the number of officially registered products in Europe remains limited (Table 1).

**Table 1:** Registered microbials for greenhouse crops in Europe (Gwynn 2014)

Classification/species	Isolate/strain	Commercial name	Target pests
Bacteria			
*Bacillus thuringiensis* subsp. *aizawai*	ABTS-1857	XenTari, Turex	Caterpillars
*B. thuringiensis* subsp. *israelensis* (serotype H-14)	AM65-52	Vectobac (Gnatrol)	Fungus gnats
*B. thuringiensis* subsp. *kurstaki*	PB 54	Belthirul	Caterpillars
*Bacillus firmus* Werner	I-1582	Bacillus firmus I-1582 WP5	Nematodes
Fungi			
*Beauveria bassiana* (Balsamo) Vuillemin	ATCC 74040, GHA	Naturalis, Botanigard	Primarily whiteflies and thrips
*Isaria fumosorosea* Wize (formerly *Paecilomyces fumosoroseus* Wize)	Apopka 97, FE9901	PreFeRal Nofly	Primarily whiteflies
*Lecanicillium muscarium* Petch (formerly *Verticillium lecanii* Zimmerman)	Ve-6	Mycotal	Primarily whiteflies
*Metarhizium brunneum* Petch (formerly *Metarhizium anisopliae* Metschnikoff*)*	Bipesco 5, F52	Taerain Met52EC	Primarily whiteflies and thrips
*Purpureocillium lilacinus* (Thom) Samson (formerly *Paecilomyces lilacinus*)	251	BioAct	Root-knot nematodes
Viruses			
*Helicoverpa armigera* nuclear polyhedrosis virus	HearNPV	Helicovex	*Helicoverpa armigera*
*Spodoptera exigua* nuclear polyhedrosis virus	Florida isolate (SeNPV-F1)	Spod-X	*Spodoptera exigua*
*Spodoptera littoralis* nuclear polyhedrosis virus	SpliNPV	Littovir	*Spodoptera littoralis*

Microbials based on subspecies of *B. thuringiensis* are registered for specific insect pests; the product based on *B. firmus* is only registered for control of nematode pests (Table 1). *Bacillus thuringiensis* forms spores that contain crystals, predominantly comprising one or more Cry and/or Cyt proteins (also known as delta-endotoxins) that lyse gut cells when consumed by susceptible insects (Gill et al. 1992). Bacterial insecticides have to be consumed in order to confer control of the pest. After ingestion, the insect gut becomes paralyzed due to the action of bacterial toxins, feeding stops and eventually the pest dies (Vachon et al. 2012). There are currently three Bt strains approved in the EU (Table 1).

The entomopathogenic fungi are a diverse assemblage of species with one thing in common: they infect and cause disease in insects and other arthropods. The majority are found within two groups: the order Hypocreales within the phylum Ascomycota, and the phylum Entomophthoromycota (Blackwell 2010; Hibbett et al. 2007; Humber 2012). In contrast with other microorganisms, entomopathogenic fungi infect their hosts by directly breaching the cuticle to enter the insect haemocoel. Their ability to invade without the requirement for ingestion is a great advantage for infecting phloem-feeding insects, such as aphids and whiteflies, which do not ingest microorganisms on the leaf surface.

*Beauveria bassiana* sensu lato*, Isaria fumosorosea* (formerly *Paecilomyces fumosoroseus*), *Metarhizium anisopliae* sensu lato. (Bischoff et al. 2009) and *Lecanicillium* (formerly *Verticillium*) species have all been reported to be effective, when sprayed in suspension, against thrips, aphids, whiteflies and weevils in greenhouse crops (de Faria and Wraight 2007; Khan et al. 2012; Skinner et al. 2012). Currently, there are five products based on entomopathogenic fungi registered in the EU, all of them based on species from the Hypocreales (Table 1). Entomopathogenic fungi in the order Entomophthorales are known for their ability to cause dramatic epizootics that rapidly reduce host populations (Pell et al. 2010). This attribute, which is more rarely seen in species from the Hypocreales, means they have the potential to be more effective biological control agents than commercially available microbials from the order Hypocreales. However, a major constraint to their augmentation as biological control agents relates to difficulties in their in vitro mass production, storage and formulation (Pell et al. 2001). This is undoubtedly the reason why the biocontrol industry has been unable to develop them as commercial microbials for augmentation (Ravensberg 2011). However, as explored further in this review, the possibility of integrating them through conservation biological control approaches seems promising.

Although a number of different types of virus infect pest arthropods, commercial microbial products are predominantly based on just one virus family, the Baculoviridae. Three baculovirus species are registered as biological control agents of lepidopteran pests of greenhouses in the EU (Table 1). Baculoviruses are generally very host specific, infecting only one or a few closely related insect species. However, two extreme examples in the context of contrasting specificity are *Spodoptera exigua* multiple nuclear polyhedrosis virus (SeMNPV), which infects only one species, *Spodoptera exigua* Hubner (the beet armyworm), and *Autographa californica* MNPV, which infects species from more than 15 families of Lepidoptera (Cory and Myers 2003). High specificity for the host makes baculoviruses good candidates for biological control, as their application does not directly affect non-target insect species.

## Combining microorganisms with arthropod natural enemies

**Fig. 1:**
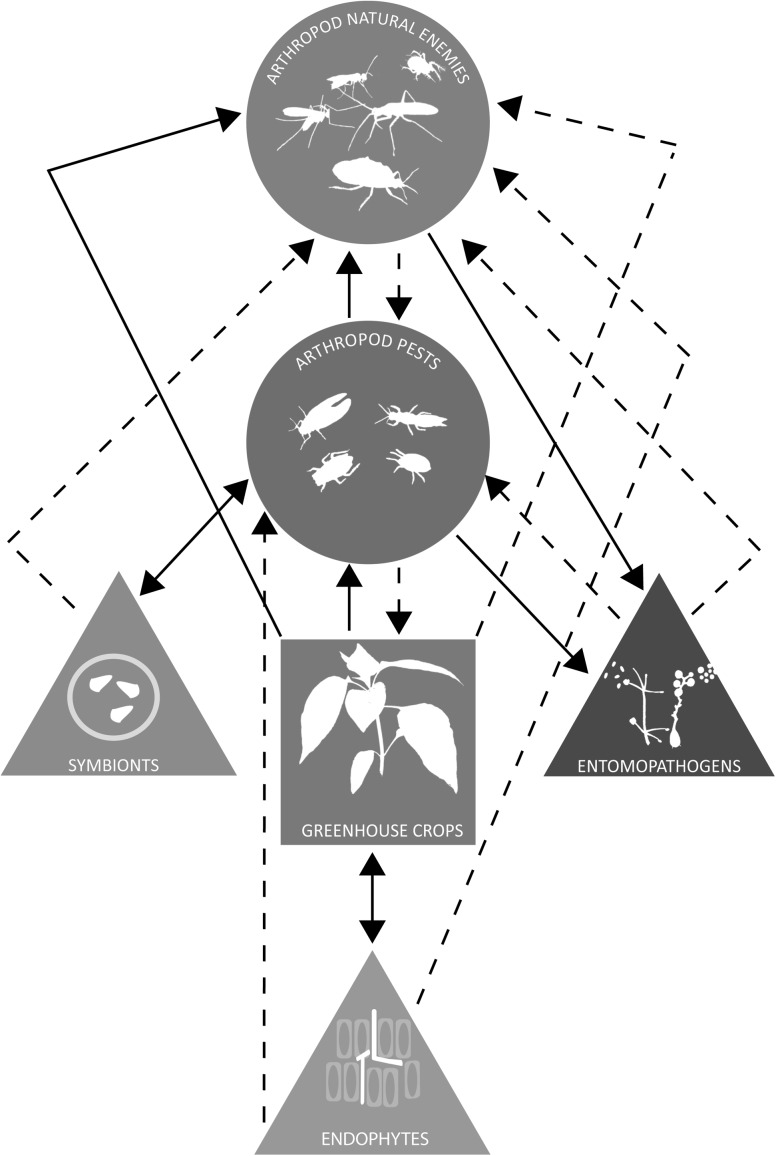
Putative interactions between crops (*square*), arthropod pests and natural enemies (*circles*), and microorganisms (*triangles*). *Dashed arrows* indicate potential negative effects, while *solid arrows* indicate potential positive effects

Use of entomopathogenic microorganisms and endophytes in greenhouse crops could have various direct and indirect effects on existing biological control systems based on arthropod natural enemies, potentially leading to both positive and negative outcomes for overall pest control (Fig. 1). Similarly, the outcome of such interactions can also be influenced by symbionts in various ways. Positive interactions represent an opportunity to enhance the efficacy of existing biological control systems.

### Microbials as a correction tool

Microbials are traditionally seen as an alternative or back-up treatment when arthropod natural enemies are unavailable or insufficiently effective (Chandler et al. 2011). For some pests, effective arthropod natural enemies are simply not available or those that are available are considered to be too expensive and hence hardly used (Table 2). Microbials could be a sustainable alternative control method in these cases. For other pests, highly effective arthropod natural enemies are commercially available, but are not effective on all crops; many natural enemies maintain a close relationship with specific plants because of their plant feeding habits or oviposition requirements (Messelink et al. 2014). For instance, western flower thrips (*Frankliniella occidentalis* Pergande), can be controlled effectively by phytoseiid predatory mites in sweet pepper, which provides pollen and nectar, but not in many ornamental plants that lack these supplemental food resources (Messelink et al. 2014). In such scenarios, the use of microbials has potential as a complementary measure in biological control programmes, as long as any potentially negative direct and indirect effects of these microorganisms are considered.

**Table 2:** Key European greenhouse pests against which arthropod natural enemies are (i) not always effective, (ii) not used because they are considered as too expensive, (iii) are unavailable or (iv) are unknown (pers. obs. by the authors of this paper)

Common name	Scientific name	Crop
Insecta: Hemiptera		
Aphids	Several species, e.g. *Myzus persicae* Sulzer *Aulacorthum solani* Kaltenbach *Aphis gossypii* Glover *Macrosiphum euphorbiae* Thomas	Vegetables and ornamentals
Armoured scales	*Diaspis boisduvalii* Signoret *Aulacaspis rosae* Bouche	Ornamentals
Mealybugs	*Planococcus citri* Risso *Pseudococcus longispinus* Targioni-Tazzetti *Pseudococcus viburni* Signoret	Vegetables and ornamentals
Tarnished plant bug Common nettle bug Common green capsid	*Lygus rugulipennis* Poppius *Liocoris tripustulatus* Fabricius *Lygocoris pabulinus* Linnaeus	Aubergine Cucumber Sweet pepper
Whiteflies	*Trialeurodes vaporariorum* Westwood *Bemisia tabaci* Gennadius	Ornamentals
Insecta: Diptera		
Cabbage root fly	*Delia radicum* Linnaeus	Radish
*Lyprauta*	*Lyprauta chacoensis* Edwards *Lyprauta cambria* Chandler	*Phalaenopsis* spp.
Insecta: Lepidoptera		
Caterpillars	several species, e.g. *Chrysodeixis chalcites* Esper *Lacanobia oleracea* Linnaeus *Duponchelia fovealis* Zeller	Vegetables and ornamentals
Insecta: Thysanoptera		
Western flower thrips	*Frankliniella occidentalis*	Ornamentals
*Echinothrips*	*Echinothrips americanus* Morgan	Ornamentals
Acari: Prostigmata		
Tomato russet mite	*Aculops lycopersici* Masse	Tomato

The ranking is based on the Class and Order of species

### Direct effects of microbials on arthropod natural enemies

Augmentative biological control is increasingly being combined with methods to conserve both the released and the naturally occurring species of arthropod natural enemies present (Messelink et al. 2014). Conservation approaches can result in the permanent presence of several interacting species of pests and natural enemies and such food webs require an ecosystem approach to manage (Janssen et al. 1998; Messelink et al. 2012). Application of additional control agents should, ideally, complement or support these persistent communities of arthropod natural enemies. The species diversity and persistence of arthropod natural enemies used to control pests in greenhouse crops strongly depend on the type of crop. This is particularly true for those natural enemies that maintain close relationships with certain host plants because of their particular plant-feeding behaviours and oviposition requirements. The predatory mirid bugs *Macrolophus pygmaeus* Rambur and *Nesidiocoris tenuis* Reuter, for example, perform well on hairy plants such as tomato and aubergine and, once present, often persist throughout the entire cropping cycle. Therefore, it is desirable that any microbial used is compatible with these persistent arthropod natural enemies, in order to conserve their populations. The most important and persistent groups of arthropod natural enemies include not only generalist predators, but also specialist species that can remain for a long time when they have reached equilibrium dynamics with their prey/host (Table 3). Such specialists include whitefly parasitoids and some spider mite predators. Sometimes achieving a stable equilibrium is attempted through deliberate releases of the pest itself (e.g. the ‘pest-in-first’ method; (Markkula and Tiittanen 1976). In such cases, it is important that the application of microbials against the same or other target pests does not disrupt the populations of arthropod natural enemies that increase resilience of cropping systems through their continuous presence.

**Table 3:** Important arthropod natural enemies in greenhouse crops that are often continuously present (Heinz et al. 2004)

Type of natural enemy: common name	Type of natural enemy: Scientific name	Crop
Generalists		
Predatory mirid bugs	*Macrolophus pygmaeus* *Nesidiocoris tenuis*	Tomato, aubergine and sweet pepper
Predatory anthocorid bugs	*Orius* spp.	Sweet pepper
Phytoseiid mites	Several species, e.g. *Neoseiulus cucumeris* Oudemans and *Amblyseius swirskii* Athias-Henriot	Sweet pepper, cucumber, aubergine, cut flowers and potted plants
Specialists		
Whitefly parasitoids	*Encarsia formosa* Gahan, *Eretmocerus eremicus* Rose & Zolnerowich, *Eretmocerus mundus* Mercet	Sweet pepper, tomato, cucumber, aubergine, poinsettia, gerbera and roses
Leafminer parasitoids	*Diglyphus isaea* Walker, *Dacnusa sibirica* Telenga	Tomato and gerbera
Predatory mites	*Phytoseiulus persimilis* Athias-Henriot *Neoseiulus californicus* McGregor	Strawberry, sweet pepper, roses and chrysanthemum

Microbials could have potential negative effects on non-target organisms, although most are considered to be highly host-specific, such as the *B. thuringiensis* subspecies, viruses of particular species of Lepidoptera and fungi from the phylum Entomophthoromycota (not registered, but often occurring spontaneously in greenhouse crops, G.J Messelink & C. Tkaczuk, pers. obs.). In contrast, entomopathogenic fungi within the order Hypocreales (Ascomycota), although isolate and species dependent, can have wider host ranges than other pathogens and could potentially kill non-target arthropod natural enemies (Roy and Pell 2000). However, these potential side effects are likely to vary significantly depending on the type of arthropod natural enemy. For example, greenhouse studies with predatory mirid bugs showed no negative effects of two commercial isolates of *B. bassiana* (GHA and ATCC 74040) on predator populations (Hamdi et al. 2011; Labbé et al. 2009). In contrast, densities of predatory *Orius* spp. were significantly reduced due to infection by the GHA isolate of *B. bassiana* (Shipp et al. 2012), although in other studies there were no or only weak side effects on *Orius* spp. predators (Hamdi et al. 2011; Pourian et al. 2011). Predatory mites generally, both in laboratory and greenhouse trials, lack vulnerability to commercial isolates of the entomopathogenic fungi *B. bassiana*, *M. brunneum* and *I. fumosorosae* (Ludwig and Oetting 2001; Vergel et al. 2011; Wu et al. 2014). In the laboratory, leafminer and whitefly parasitoids can be highly susceptible (30–70 %) to *B. bassiana* (Shipp et al. 2003), but in greenhouse trials only low levels of infection were observed in whitefly parasitoid populations (Labbé et al. 2009; Shipp et al. 2012). In a recent study, the parasitoid *Trybliographa rapae* Westwood was shown to be susceptible to *B. bassiana* and *M. brunneum*, but further experiments showed that it was also able to recognize and avoid *M. brunneum* suggesting these two biological control agents are compatible (Rännbäck et al. 2015). Similarly, a new study has shown that the mortality of different arthropods for the control of the western flower thrips ranged from 3 to 61 % when combined with entomopathogenic fungi in laboratory tests (Saito and Brownbridge 2016). However, their results also indicate that compatibility and overall increased effects are observed when both biological control agents are applied. Therefore, the majority of studies suggest that microbials are compatible with arthropod natural enemies, but caution should be practiced with the application of entomopathogenic fungi with broad host ranges.

### Indirect effects of microbials on natural enemies

The application of microbials to crops can have unexpected effects on arthropod natural enemies through changes in behaviour of the pests, the arthropod natural enemies or through changes in pest densities or pest diversity (Roy and Pell 2000; Roy et al. 2006). This latter aspect is particularly relevant for generalist predators that feed on multiple pest species. Changes in the density of one pest species due to the application of a target-specific microbial can indirectly affect other pest species, simply because of the availability of food or because the predators perform better on mixed diets of pests (Messelink et al. 2008, 2010; Muñoz-Cárdenas et al. 2014). Such predator-mediated effects should be taken into account when applying microbials against a specific pest species.

Other negative interactions can occur as a result of avoidance. Predatory bugs may, in some cases, avoid predating on infected prey or plants treated with microbials, which could increase their prey searching time and reduce predation rates (Labbé et al. 2009; Meyling and Pell 2006; Pourian et al. 2011). Several parasitoid species seem to be able to detect whether their host is infected by an entomopathogenic fungus, such as the whitefly parasitoid *Encarsia formosa* (Fransen and van lenteren 1993) and the aphid parasitoid *Aphelinus asychis* Walker (Mesquita and Lacey 2001). This is positive for the survival of the parasitoid’s offspring, but at the same time may increase searching time and thereby reduce efficacy. Other parasitoid species are not able to detect infected living hosts, as was reported for *Aphidius ervi* Haliday and aphids infected by *Pandora neoaphidis* Remaudiere & Hennebert (Baverstock et al. 2005). This could have a tremendous negative effect on the efficacy of this parasitoid, as parasitoids developing in infected aphids will not develop into adults. Natural enemies may also predate on infected hosts, thereby reducing the efficacy of microbial applications (Pell et al. 1997; Roy et al. 2008). All these complex interactions may lead to nonadditive effects of combined treatments. Sometimes it may be possible to avoid this by adapting the timings of microbial applications and releases of arthropod natural enemies in order to minimize potential negative effects. For example, parasitoids might be more effective when their release time after a microbial application is long enough to allow the separation of entomopathogen-infected hosts from uninfected hosts (Fransen and van lenteren 1993).

Positive, potentially synergistic, effects can also occur when the arthropod natural enemies enhance the dispersal of entomopathogens. Down et al. (2009) showed enhanced control of aphids when conidia of the entomopathogenic fungi *Lecanicillium longisporum* Zimmerman were disseminated by *Orius laevigatus* Fieber. Furthermore, the presence of coccinellid predators increased the proportion of aphids becoming infected by the fungal pathogen *P. neoaphidis* due to increased transmission (Roy et al. 2001; Wells et al. 2011). Some predators may further increase dispersal of conidia of aphid pathogens by inducing the production of winged morphs (Müller et al. 2001). Predators and parasitoids may also facilitate infection by microbials when their foraging activity increases movement of the pest, increasing the likelihood that pathogen propagules come into contact with their hosts (Roy and Pell 2000).

Finally, microbials and predators may act synergistically when sublethal doses of the pathogen make the pest more vulnerable to predation, perhaps due to reduced defence responses or through extended larval development times. Such effects have been observed for Colorado potato beetle (*Leptinotarsa decemlineata* Say) larvae that were more successfully attacked by the predator *Perillus bioculatus* Fabricius after treatment with sublethal doses of *B. thuringiensis* (Cloutier and Jean 1998). Similarly, microbials can make pests more vulnerable to some biopesticides such as neem (Mohan et al. 2007). To our knowledge, there are no studies that categorically demonstrate synergistic or additive effects from combined treatments of microbials and predators in greenhouse crops, but, based on the studies mentioned, this could be a promising area for future research. This might be particularly interesting for pests, such as western flower thrips (Almeida and Janssen 2012) and some caterpillars, e.g. *C. chalcites* (G.J. Messelink pers. obs.), that have strong defensive responses to their arthropod natural enemies.

### Increasing efficacy with new formulation and application techniques

The success of a biological control programme with microbials depends on how much of the agent used reaches the target pests. For this reason, the way that microbials are formulated and applied is particularly important because the pathogen must be able to survive under greenhouse conditions but must also reach the intended pest (Lohse et al. 2015). Formulation plays an important role in delivering the pathogen to the target environment. Formulated microbials are typically prepared as technical concentrates, wettable powders or oil dispersions (de Faria and Wraight 2007). Some adjuvants and other ingredients can improve the persistence of microbials in the environment by protecting them from inactivation by sunlight (Reddy et al. 2008; Shapiro 1992). The most promising technology to date is encapsulation within a matrix, which provides a stable microenvironment and protects the microbial from biotic and abiotic stress factors (contamination, soil antagonists, temperature, dryness, UV light, mechanical stress) (Vemmer and Patel 2013). This extends the shelf life and maintains metabolic activity for prolonged time periods not only during storage but also after application resulting in a reduction in the dose and number of applications required.

Traditionally microbials have been applied in a similar way to pesticides (Chandler et al. 2011), with associated problems of how to ensure that microbial formulations survive passage through spraying devices and the potential negative impacts of massive inundative applications of microbials on beneficial invertebrates, such as arthropod natural enemies and pollinators. To avoid massive applications of microbials, techniques for new applications have been developed. One such alternative option is seed treatment. Keyser et al. (2014) found that when seeds were coated with *Metarhizium* species, the fungi were able to disperse via the roots and induce infections in insects feeding on the roots. Specifically, in laboratory and greenhouse experiments, *Tenebrio molitor* Linnaeus larvae were exposed to roots of wheat plants that had been grown from seeds inoculated with conidia of either *M. brunneum* or *M. robertsii*. All four *Metarhizium* isolates tested maintained pathogenicity towards *T. molitor* larvae for up to 4 weeks after being dispersed by roots through both artificial substrates and nonsterile soil. Based on these results, the authors proposed that plant–root associations improved the mobility of entomopathogenic fungi in the soil and increased their likelihood of encountering susceptible insect hosts. Another way to achieve this plant–root colonization is the inoculation of growing substrates with *Metarhizium sp.,* which appeared very effective in controlling black vine weevil larvae, *Otiorhynchus sulcatus* F., up to a year after application (Bruck 2005, 2010). Adding entomopathogens to growing media can also be useful for controlling soil-dwelling stages of pests that do not attack roots, such as pupae of western flower thrips (Ansari et al. 2008). With further research, treating seeds or growing media with entomopathogenic fungi has the potential to be successfully applied in a wide range of greenhouse conditions.

Another interesting technique to achieve more targeted application of microbials is the auto-dissemination approach, which aims to promote the transmission of infective pathogen propagules within and between insect populations. For example, the ability of bumblebees to disseminate conidia of *B. bassiana* from hive-mounted dispensers to greenhouse crops shows potential for control of whiteflies, thrips and *Lygus* species on tomatoes and sweet pepper (Al-mazra’awi et al. 2006; Kapongo et al. 2008). Arthropod natural enemies may also contribute to the dispersal of microbials as described previously (Roy and Pell 2000). Promising results were achieved with predatory mites; when dusted with *B. bassiana* prior to their release, *A. swirskii* and *N. californicus* increased mortality in the pest *Diaphorina citri* Kuwayama, while maintaining low mortality of their own species (Zhang et al. 2015).

Auto-dissemination can be targeted even more specifically by using devices that contain both entomopathogens and attractive species-specific semiochemicals; the pests enter the device in response to the semiochemical, become contaminated by the pathogen (which will eventually cause their death), exit from the device and spread the infection amongst conspecifics (Baverstock et al. 2010; Lacey et al. 2015; Vega et al. 1995, 2007). To increase the chances of infection, traps are baited with highly attractive semiochemicals that indicate the presence of food, mates or oviposition sites. This method is often known as ‘lure and infect’ as opposed to ‘lure and kill’ because the pests do survive for a short time during which they interact with other individuals promoting transmission and the development of epizootics. Combinations of pheromone lures and entomopathogenic fungi have been successfully used to control insects such as bark beetles (*Ips typographus* Linnaeus), weevils (*Cylas formicarius* Fabricius*, Cosmopolites sordidus* Germar), moths (*Plutella xylostella* Linnaeus), greenbugs (*Plautia crossota* stali Scot), thrips (*Megalurothrips sjostedti* Trybom) and aphids (*Phorodon humuli* Schrank) under field conditions (Hartfield et al. 2001; Kreutz et al. 2004; Lopes et al. 2014; Mfuti et al. 2016; Pell et al. 1993; Roditakis et al. 2008; Tinzaara et al. 2007; Tsutsumi et al. 2003; Vickers et al. 2004; Yasuda 1999). However, the use of pheromones has limitations because in many cases aggregation pheromones, which attract both sexes, are unknown and sex pheromones tend to be sex-specific i.e. only one sex is attracted and controlled. Nevertheless, other olfactory cues such as food and host odours also have potential as attractants (Klein and Lacey 1999; Lecuona et al. 2005; Renn et al. 1999). For instance, combining the codling moth granulovirus (CpGV) with apple-associated yeasts increased the mortality of codling moth (*Cydia pomonella* Linnaeus) under laboratory and field conditions (Knight and Witzgall 2013). The potential for combining microbials with semiochemicals for pest control under greenhouse conditions is evident, especially considering that key greenhouse pests such as thrips, moths and aphids are susceptible to pathogens and there is a vast amount of knowledge concerning their chemical communication already available (Elimem et al. 2014; Furlong and Pell 2001; Niassy et al. 2012; Pickett et al. 1992). However, for lure and infect strategies to be successful in greenhouse crops, more research is required to develop and optimize lure and infect devices and to evaluate such systems under greenhouse conditions.

### Conservation of microbials in greenhouse crops

Conservation biological control relies on modification of the environment or management practices, to protect and encourage natural enemies that are already present within the system (Landis et al. 2000). Various methods are used for conservation of arthropod natural enemies in greenhouse crops. For example, the use of banker plants that provide alternative hosts or food resources (Messelink et al. 2014), but conservation of microbials has not received much attention. This is probably because microbials have traditionally been used to react to outbreaks of pests rather than as a preventive control measure. However, in the review paper of Pell et al. (2010) several methods to conserve fungal entomopathogens and stimulate natural epizootics are suggested. These include: (1) preventing intense soil disturbance as many fungal entomopathogens have at least part of their life cycle in the soil, (2) preventing frequent use of fungicides, (3) encouraging non-pest alternative hosts and (4) encouraging other arthropods for dispersal of conidia. Although these practices are not widely used in greenhouse crops, some organic growers are exploring the use of banker plants to encourage non-pest aphids infected by entomophthoralean fungi and thereby induce natural epizootics of these fungi (Messelink & Dinu, personal observations). There are several options for the use of non-pest aphids as reservoirs for these specialized aphid pathogens; for example the grain aphid, *Sitobion avenae* (Shah et al. 2004), which is commonly used as a banker plant system for aphid parasitoids (Huang et al. 2011) could also support multiplication of specialist aphid pathogens. Because these aphids are adapted to monocotyledonous plants, they cannot survive on dicotyledonous crops grown in greenhouses, thus using such aphids poses no risk and is relatively simple. Furthermore, the banker plant itself may contribute to the survival and persistence of entomopathogens. Hairy leaves may provide a better microclimate and leaf waxiness could increase adhesion and germination of conidia on the insect cuticle (Cory and Hoover 2006). Besides the provision of alternative hosts on banker plants, companion plants in general could be considered to support the survival of microbials. An additional benefit of these banker and companion plants could be that they provide pollen and nectar for arthropod natural enemies and bees that could simultaneously aid dispersal of entomopathogens from the banker to the crop as they move back and forth between them, but this idea requires further elaboration.

Uniquely, greenhouses also provide the opportunity to use UV-blocking screens that can increase the survival of microbials. Several studies have shown the damaging effects of UV light on entomopathogenic fungi and viruses (Braga et al. 2001, 2002; Cory and Hoover 2006) and that survival of fungal entomopathogens is better in greenhouses with UV-blocking screens than in greenhouses without screens (Diaz and Fereres 2007). Not only plastic covers, but also greenhouse glass can partly or totally filter out UV-B wavelengths (Hemming et al. 2012), which will be beneficial for the survival of entomopathogens. Finally, by combining conservation tools with the selection of microbial isolates with particular traits that make them easier to conserve after application (such as persistence), could increase their biological control potential (Cory and Franklin 2012).

### Balancing climatic adaptation for microbials and arthropod natural enemies

Greenhouse systems have been developed to protect crops from unfavourable environmental conditions and, depending on the geographical area, these systems range from low-tech plastic tunnels to high-tech glasshouses (van Henten et al. 2006). Globally, various technologies for managing temperature, humidity, light and CO_2_ levels are increasingly used to optimize the environment for crop growth (Montero 2011). The most advanced technologies are mainly driven by the need to save energy and reduce fossil fuel consumption (Vadiee and Martin 2014). This need has resulted in new concepts such as the closed greenhouse system where cooling by ventilation is replaced by mechanical cooling and excess solar energy is collected and stored to be reused to heat the greenhouse (De Gelder et al. 2012). Implementing such new techniques enhances the possibilities to completely manage the greenhouse climate. Interestingly, these new techniques also offer new opportunities to temporarily adapt the greenhouse climate for other purposes, such as optimizing pest control with microbials. For example, increasing greenhouse humidity levels significantly increased pest control with *B. bassiana* (Shipp et al. 2003). Such decisions obviously need to be considered carefully, as some climatic benefits for microbials may have detrimental effects on crop growth, arthropod natural enemies or may favour some plant diseases. Also, it needs to be mentioned that the relative humidity in the microclimate can be significantly different to the ambient conditions. For example, high humidity levels at the leaf surface may be sufficient for infection by entomopathogenic fungi, even when the ambient relative humidity levels are lower. One important advance in greenhouse climate management is the development of temperature integration regimes for reducing energy consumption, which allow higher temperatures during the day to be tolerated and compensated for by lower temperatures during the night (Körner and Challa 2003). Experiments showed that an extra benefit of this regime could be a reduced influx of thrips from outside into the greenhouse, because vents are opened less frequently than when traditional climate management is used (Jakobsen et al. 2006). The lower temperatures at night and in the early hours of the morning may also increase efficacy of Entomophthorales (Milner et al. 1984), but further research is needed to determine whether the higher day temperatures might be detrimental for the survival of these fungi and other microbials. Potential negative effects on arthropod natural enemies also require further evaluation. For example, lower night temperatures might be detrimental for arthropod natural enemies that are night active, such as the aphid predatory midge *Aphidoletes aphidimyza* Rondani, which requires a minimum temperature to be flight-active (Markkula et al. 1979). Diurnal temperature ranges of more than 15 °C could also be detrimental for biological control of spider mites by *Phytoseiulus persimilis* (Vangansbeke et al. 2015).

Overall it is a huge advantage to have the opportunity to adapt the greenhouse climate in order to optimize microbial efficacy, particularly when the potential damage by the target pest is larger than the potential crop losses that may occur due to less favourable climatic conditions, plant diseases or reduced efficacy of some arthropod natural enemies. Not only temperature and humidity, but also artificial light in greenhouses might influence pest control (Johansen et al. 2011), but the effects on microbials are relatively unknown. While it is known that the survival of fungal conidia is significantly reduced when exposed to UV-A and UV-B irradiation (Yao et al. 2010), and as we mentioned earlier, there are opportunities to enhance pest control by fungal microbials by using UV-blocking covers (Costa et al. 2001). This technique seems to be compatible with natural enemies, as no negative effects have been found to date (Dáder et al. 2015; Doukas and Payne 2007). Hence, there are several methods now available to adapt the greenhouse environment and these warrant further study. For example, which combinations of artificial light and greenhouse climatic conditions are optimal for pest control with microbials and arthropod natural enemies? It is not only the direct environmental effects on microbials, but also the indirect effects through changes in pest behaviour that could affect these results (Johansen et al. 2011).

### Endophytes for pest control

A relatively new field of research in pest control with microorganisms aims to make use of fungal species that act as endophytes. The term endophyte refers to fungi and bacteria that develop within plant tissues without causing any conspicuous symptoms of disease in the plant (Wilson 1995). Besides the class II fungal endophytes (sensu Rodriguez et al. (2009)), which have a broad host plant range and are able to survive in the environment without a host, entomopathogenic fungi are also known to colonize plant tissues (see recent review by Vidal and Jaber (2015)).

So far several studies provided evidence that entomopathogenic fungi are able to confer at least partial resistance to their host plants when colonizing plant tissues. For instance, prior inoculation of tomato plants with the endophytic fungus *Acremonium strictum* Gams resulted in a significant decrease in the performance of pests such as greenhouse whiteflies (*Trialeurodes vaporariorum* (Westwood)) and the cotton bollworm (*Helicoverpa armigera* (Hübner)) compared with noninoculated plants (Jallow et al. 2004; Vidal 1996). Other examples include control of the diamondback moth, *Plutella xylostella* (Linnaeus), on cabbage inoculated with an endophytic strain of *A. strictum* (Raps and Vidal 1998); reduction in populations of *Aphis gossypii* Glover on squash plants previously inoculated with an endophytic isolate of *Fusarium oxysporum* Snyder & Hanssen (Martinuz et al. 2012); and retarded development of *Thrips tabaci* Lindeman on onions inoculated with several species of fungal endophytes (Muvea et al. 2014). The underlying mechanisms mediating the effects on herbivorous insects remain, however, to be investigated in detail. The effects were hypothesized to be due to metabolic products produced by the endophytic fungi or by plant metabolites induced by their presence. Compounds produced by Class II fungal endophytes are mainly extracellular enzymes like proteases, lipases and cellulases, and metabolites like beauvericin, oosporein, fumonisin, harzianolide, butenolide and fusaric acid (Gurulingappa et al. 2010, 2011; Ownley et al. 2008; Vega et al. 2009).

However, some natural enemies also feed on the plants harbouring endophytic fungi and could attack herbivorous insects that may be experiencing negative effects due to fungal endophytes. This appeared to be the case for the omnivorous predatory bug *M. pygmaeus* on tomato plants previously inoculated with a nonpathogenic isolate of *F. oxysporum* (Messelink et al., unpublished data). Surprisingly, this endophyte still enhanced pest control by deterring the predator from feeding on the plant, thereby increasing its feeding activity on the prey. The parasitoid *Bracon hebetor* Say, when parasitizing larvae of *Spodoptera litura* Fabricius feeding on cauliflower inoculated by *Aspergillus* spp., experienced prolonged development time of the larvae and reduced parasitism rates by the adults (Kaur et al. 2015). Thus, the use of Class II endophytic fungi for control of herbivorous pests in greenhouses needs to be tested on a case by case basis for each plant-herbivore-natural enemy-interaction.

One of the most striking recent discoveries with regard to endophytic fungi is that several, if not all, isolates of entomopathogenic fungi can also act as endophytes. Most research on entomopathogenic fungi has focused on their virulence and the mechanisms they use to infect the host directly through the cuticle (Vega et al. 2008). However, endophytic isolates of *B. bassiana*, *M. brunneum* and *L. muscarium* that are active within plants have shown potential to control insects such as the corn borer *Ostrinia nubilalis* Hübner (Bing and Lewis 1991); the coffee berry borer *Hypothenemus hampei* Ferrari (Posada et al. 2007); the banana weevil *Cosmopolites sordidus* (Akello et al. 2008); the aphids *Acyrthosiphon pisum* Harris and *Aphis fabae* Scopoli (Akello and Sikora 2012); the American bollworm *H. armigera* (Qayyum et al. 2015) and the pea leafminer *Liriomyza huidobrensis* Blanchard (Akutse et al. 2013).

Bacterial endophytes have been studied mainly for their potential as plant growth promoters and for their ability to induce systemic resistance to plant pathogens (Kavino et al. 2007; Ryan et al. 2008; Sturz et al. 2000). Direct control of pests has not been reported for bacterial endophytes. Indirect effects caused by the induction of systemic resistance and by changes to the chemical profile of plants have been suggested as having the potential to influence the performance of some pests (Kloepper and Ryu 2006; Valenzuela-Soto et al. 2010).

The fact that bacterial and fungal endophytes might prime plants for resistance to both pests and diseases makes them formidable as biological control agents. For example, endophytic isolates of *F. oxysporum* have the ability to suppress aphids and also nematodes, Oomycota and other plant diseases (Fuchs et al. 1997; Hallmann and Sikora 1996; Kim et al. 2007; Martinuz et al. 2012). The use of endophytes that decrease performance by extending pest developmental time might also provide an opportunity to increase the impact of arthropod natural enemies in greenhouses. Irrespective of the mechanism, the longer it takes the pest to develop and complete its life cycles, the more exposed it is to parasitoids and predators. Finally, the use of endophytes introduced at an early stage of plant development (such as seeds coated with endophytes) circumvents the intrinsic problems of microbial use such as exposure to detrimental environmental factors, competition with other microorganisms and the challenge of synchronizing microbials with herbivore presence. However, entomopathogenic fungi are obviously not able to fully colonize all tissues of fast growing plants (Behie et al. 2015), resulting in several plant parts not being protected, and the interactions between specific entomopathogenic fungal isolates, the plant cultivar and the growing media (Tefera and Vidal 2009) seems to influence the extent to which these fungi can mediate herbivore preference and performance. Climate regimes in greenhouses may be beneficial for most fungi because they provide specific environmental conditions (constant higher temperatures and humidity), and most probably are also favourable for endophytic establishment in plants. However, isolates specifically adapted to these conditions also need to be selected to increase colonization rates of plant tissues. Thus, a screening process to find the optimal combination of the different agents is mandatory for the implementation of this strategy. The use of endophytic organisms as biological control agents might become even more complicated as a result of the findings of Yan et al. (2015) who demonstrating a ‘fight for niches’ within single plants, for regions not yet colonized by other endophytic organisms. Endophytes are known for their complex metabolic capabilities (Gutierrez et al. 2012) and some of the compounds they produce which are aimed at maintaining a multipartite symbiosis with competing microorganisms (Schulz et al. 2015), might also have adverse effects when consumed by humans. Thus, the method and timing of the inoculation of the crop plant, and the previous history of microbial colonization events, plays an important role in the potential for success with this strategy. Some of these problems may be addressed by the development of specific formulations for endophytes that enhance colonization rates and provide a headstart for these organisms within seedlings. However, these aspects need to be rigorously tested to gain a better understanding of the potential of microorganisms as biological control agents.

### Insect-associated microorganisms

In recent years, it has been increasingly recognized that microbial symbionts affect the ecology, life history and evolution of their arthropod hosts in many different ways (Zchori-Fein and Bourtzis 2012). A lot of the key greenhouse pests are exclusively sap feeders, and like other insects with limited diets, harbour a wide range of microbial associates (Zchori-Fein and Bourtzis 2012). All phloem and xylem-feeding pests harbour maternally inherited, intracellular, nutritional bacterial symbionts; these are ‘obligate’ or ‘primary’ symbionts that provide their hosts with essential nutrients missing in the plant sap and are often restricted to specialized organs called bacteriomes. In addition, sap-feeders also commonly harbour facultative or ‘secondary’ symbionts which are involved in nearly all aspects of their host’s biology, including protecting them against natural enemies (Oliver et al. 2003; Teixeira et al. 2008) and heat stress (Montllor et al. 2002); their performance on crops (Hosokawa et al. 2007); and in increasing their ability to transmit plant viruses (Gottlieb et al. 2010; Van den Heuvel et al. 1994). These ‘secondary’ symbionts also manipulate the reproduction of their hosts in ways that increase the frequency of female hosts becoming infected (e.g. Werren et al. (2008)). Furthermore, sap-feeders are usually associated with extracellular gut microbes, which may have nutritional or metabolic functions (Kikuchi et al. 2011; Werren et al. 2008). Although studies on host-microbial symbionts in insects have focused on bacteria, archaeal and fungal symbioses have also been reported (Gibson and Hunter 2010).

Zindel et al. (2011) summarized the various ways by which symbiotic microorganisms may dramatically affect all phases of augmentative biological control, from the mass rearing of natural enemies to their efficiency in the field. For example, symbiotic bacteria may induce cytoplasmic incompatibility, resulting in sterile eggs when a symbiont-infected female mates with an uninfected male. A mixed population of symbiont-infected and noninfected individuals would thus reproduce more slowly than one in which all members carried the symbiont or where all members were symbiont-free. In addition, symbiotic microorganisms can increase or decrease the survivorship of an insect host under extreme environmental conditions or influence the transmission capacity of disease-vectoring arthropods (Zindel et al. (2011) and refs there in).

Amongst their other phenotypes, insect microbial associates may protect their hosts against natural enemies such as parasitoids, bacteria, viruses and fungi, and these ‘defensive’ interactions have been thoroughly reviewed (Oliver et al. 2014). Most relevant to this review are examples concerning greenhouse pests with no effective biological control solutions (Table 2), such as aphids and whiteflies. The pea aphid, *Acyrthosiphon pisum*, has four different symbionts belonging to the bacterial genera *Regiella, Rickettsia, Rickettsiella* and *Spiroplasma*, which induce defences against the entomopathogenic fungus *P. neoaphidis*. The presence of these distantly related symbionts in aphids reduces both their susceptibility to *P. neoaphidis* infection and sporulation on *P. neoaphidis*-infected aphid cadavers (Łukasik et al. 2013). Although information on the symbionts of aphid species that are greenhouse pests is rather scarce, bacteria such as *Rickettsia* have been reported from the cotton aphid, *A. gossypii*, and the potato aphid *M. euphorbiae*, although the effects of these symbionts on their phenotypes has not been determined. Recently Hendry et al. (2014) showed that bacterial symbionts from the genus *Rickettsia* greatly reduced the efficacy of the entomopathogenic bacterium *Pseudomonas syringae* Van Hall against the sweet potato whitefly, *B. tabaci*. There is every reason to assume that similar symbiotic interactions may occur in other insects colonizing greenhouse crops, and this needs to be taken into account when microbials are produced and applied for pest control in greenhouses and other crop systems.

Symbionts are, so far, not deliberately added to biological control systems, but this might be a field for future research. For example, aphid biotypes that contain particular symbionts might be used to select for parasitoids that are adapted to these symbionts, in order to enhance their performance in the field.

## Conclusions and recommendations

Although greenhouse crop production seems ideal for the use of biological control agents, experience has taught us that there are a range of interactions that must be considered when incorporating organisms into a closed system. For decades, research focussed on understanding the tritrophic interactions between plants, herbivores and their arthropod natural enemies (Kennedy 2003; Price et al. 1980; Vet and Dicke 1992). However, it is now clear that tritrophic interactions are influenced by all the other components of the ecosystem, and as a result, research has shifted into understanding crop-pest management from the perspective of multitrophic interactions (Van der Putten et al. 2001). As we have shown here, the combined use of microbial biological control with the use of arthropod natural enemies must take into account direct and indirect effects on each side.

Understanding the complexity of ecological interactions between different types of biological control agents is itself a subject that requires further research. In terms of biological control, studies need to focus on unveiling direct and indirect effects of the application of microbial biological control agents and microbial communities within insects (symbionts) and plants (endophytes) on arthropod natural enemies. Although most cases show evidence of compatibility between different types of agents, it is necessary to pinpoint the essential conditions that ensure the success of their combination. We consider that the following research areas should be prioritized: (1) optimizing the efficacy of the existing and new microbial products and (2) determining how to combine both microbial and arthropod natural enemies with available technologies. Optimization of microbial efficacy is an ongoing goal achieved by new formulation and conservation techniques and by selection of specific and virulent isolates with the most desirable characteristics to be effective and persist under greenhouse conditions. Combining microbials with the existing and new technologies is already promising since greenhouse climate control and new delivery methods are already available. The use of endophytes and manipulation of symbionts to improve control of pests could also represent elegant solutions to many of the current problems with microbials, but further research is required.

Understanding of the ecological consequences of using microbials in combination with other biological control agents needs closer examination, especially for organic greenhouse cropping systems that have evolved into complex ecosystems with persistent populations of multiple arthropod natural enemy species. To our surprise, there are only a limited number of studies involving microbials and arthropod natural enemies under greenhouse conditions, confirming that this area is fertile ground for research. In our opinion, such studies deserve more attention as they may help to identify complementary and synergistic interactions between microbials and arthropod natural that increase the opportunities to enhance and further develop biological pest control.

## Authors contribution

All authors contributed to the writing of this review. FG and GM edited and finalized the manuscript.

## References

[CR1] Akello J, Sikora R (2012). Systemic acropedal influence of endophyte seed treatment on *Acyrthosiphon pisum* and *Aphis fabae* offspring development and reproductive fitness. Biol Control.

[CR2] Akello J, Dubois T, Coyne D, Kyamanywa S (2008). Endophytic *Beauveria bassiana* in banana (Musa spp.) reduces banana weevil (*Cosmopolites sordidus*) fitness and damage. Crop Prot.

[CR3] Akutse KS, Maniania NK, Fiaboe KKM, Van den Berg J, Ekesi S (2013). Endophytic colonization of *Vicia faba* and *Phaseolus vulgaris* (Fabaceae) by fungal pathogens and their effects on the life-history parameters of *Liriomyza huidobrensis* (Diptera: Agromyzidae). Fungal Ecol.

[CR4] Al-mazra’awi MS, Shipp L, Broadbent B, Kevan P (2006). Biological control of *Lygus lineolaris* (Hemiptera : Miridae) and *Frankliniella occidentalis* (Thysanoptera : Thripidae) by *Bombus impatiens* (Hymenoptera : Apidae) vectored *Beauveria bassiana* in greenhouse sweet pepper. Biol Control.

[CR5] Almeida Â, Janssen A (2012) Juvenile prey induce antipredator behaviour in adult predators. Exp Appl Acarol, pp 1–8 10.1007/s10493-012-9601-6 PMC3557378 22923143

[CR6] Ansari MA, Brownbridge M, Shah FA, Butt TM (2008). Efficacy of entomopathogenic fungi against soil-dwelling life stages of western flower thrips, Frankliniella occidentalis, in plant-growing media. Entomol Exp Appl.

[CR7] Baverstock J, Alderson PG, Pell JK (2005). Pandora neoaphidis transmission and aphid foraging behaviour. J Invertebr Pathol.

[CR8] Baverstock J, Roy HE, Pell JK (2010). Entomopathogenic fungi and insect behaviour: from unsuspecting hosts to targeted vectors. Biocontrol.

[CR9] Behie SW, Jones SJ, Bidochka MJ (2015). Plant tissue localization of the endophytic insect pathogenic fungi Metarhizium and Beauveria. Fungal Ecol.

[CR10] Bing LA, Lewis LC (1991). Suppression of *Ostrinia nubilalis* (Hübner) (Lepidoptera: Pyralidae) by endophytic *Beauveria bassiana* (Balsamo) Vuillemin. Environ Entomol.

[CR11] Bischoff JF, Rehner SA, Humber RA (2009). A multilocus phylogeny of the *Metarhizium anisopliae* lineage. Mycologia.

[CR12] Blackwell M (2010). Fungal evolution and taxonomy. Biocontrol.

[CR13] Braga GUL, Flint SD, Miller CD, Anderson AJ, Roberts DW (2001). Both solar UVA and UVB radiation impair conidial culturability and delay germination in the entomopathogenic fungus *Metarhizium anisopliae*. Photochem Photobiol.

[CR14] Braga GUL, Rangel DEN, Flint SD, Miller CD, Anderson AJ, Roberts DW (2002). Damage and recovery from UV-B exposure in conidia of the entomopathogens *Verticillium lecanii* and *Aphanocladium album*. Mycologia.

[CR15] Bruck DJ (2005). Ecology of *Metarhizium anisopliae* in soilless potting media and the rhizosphere: implications for pest management. Biol Control.

[CR16] Bruck DJ (2010). Fungal entomopathogens in the rhizosphere. Biocontrol.

[CR17] Chandler D, Bailey AS, Tatchell GM, Davidson G, Greaves J, Grant WP (2011). The development, regulation and use of biopesticides for integrated pest management. Philos Trans R Soc B-Biol Sci.

[CR18] Cloutier C, Jean C (1998). Synergism between natural enemies and biopesticides: a test case using the stinkbug *Perillus bioculatus* (Hemiptera : Pentatomidae) and *Bacillus thuringiensis**tenebrionis* against Colorado potato beetle (Coleoptera : Chrysomelidae). J Econ Entomol.

[CR19] Cory JS, Franklin MT (2012). Evolution and the microbial control of insects. Evol Appl.

[CR20] Cory JS, Hoover K (2006). Plant-mediated effects in insect-pathogen interactions. Trends Ecol Evol.

[CR21] Cory JS, Myers JH (2003). The ecology and evolution of insect baculoviruses. Annu Rev Ecol Evol Syst.

[CR22] Costa HS, Robb KL, Wilen CA (2001). Increased persistence of *Beauveria bassiana* spore viability under high ultraviolet-blocking greenhouse plastic. HortScience.

[CR23] Dáder B, Plaza M, Fereres A, Moreno A (2015). Flight behaviour of vegetable pests and their natural enemies under different ultraviolet-blocking enclosures. Ann Appl Biol.

[CR24] de Faria MR, Wraight SP (2007). Mycoinsecticides and mycoacaricides: a comprehensive list with worldwide coverage and international classification of formulation types. Biol Control.

[CR25] De Gelder A, Dieleman JA, Bot GPA, Marcelis LFM (2012). An overview of climate and crop yield in closed greenhouses. J Hortic Sci Biotechnol.

[CR26] Diaz BM, Fereres A (2007). Ultraviolet-blocking materials as a physical barrier to control insect pests and plant pathogens in protected crops. Pest Technol.

[CR27] Doukas D, Payne CC (2007). Effects of UV-blocking films on the dispersal behavior of *Encarsia formosa* (Hymenoptera : Aphelinidae). J Econ Entomol.

[CR28] Down RE, Cuthbertson AGS, Mathers JJ, Walters KFA (2009). Dissemination of the entomopathogenic fungi, *Lecanicillium longisporum* and *L. muscarium*, by the predatory bug, *Orius laevigatus*, to provide concurrent control of *Myzus persicae*, *Frankliniella occidentalis* and *Bemisia tabaci*. Biol Control.

[CR29] Elimem M, da Silva JAT, Chermiti B (2014). Double-attraction method to control *Frankliniella occidentalis* (Pergande) in pepper crops in Tunisia. Plant Protect Sci.

[CR30] Fransen JJ, van lenteren JC (1993). Host selection and survival of the parasitoid *Encarsia formosa* on greenhouse whitefly, *Trialeurodes vaporariorum*, in the presence of hosts infected with the fungus *Aschersonia aleyrodis*. Entomol Exp Appl.

[CR31] Fuchs JG, MoenneLoccoz Y, Defago G (1997). Nonpathogenic *Fusarium oxysporum* strain Fo47 induces resistance to fusarium wilt in tomato. Plant Dis.

[CR32] Furlong MJ, Pell JK (2001). Horizontal transmission of entomopathogenic fungi by the diamondback moth. Biol Control.

[CR33] Gibson CM, Hunter MS (2010). Extraordinarily widespread and fantastically complex: comparative biology of endosymbiotic bacterial and fungal mutualists of insects. Ecol Lett.

[CR34] Gill SS, Cowles EA, Pietrantonio PV (1992). The mode of action of *Baccilus thuringiensis* endotoxins. Annu Rev Entomol.

[CR35] Glare T (2012). Have biopesticides come of age?. Trends Biotechnol.

[CR36] Gottlieb Y (2010). The transmission efficiency of tomato yellow leaf curl virus by the whitefly *Bemisia tabaci* is correlated with the presence of a specific symbiotic bacterium species. J Virol.

[CR37] Gurulingappa P, Sword GA, Murdoch G, McGee PA (2010). Colonization of crop plants by fungal entomopathogens and their effects on two insect pests when in planta. Biol Control.

[CR38] Gurulingappa P, McGee PA, Sword G (2011). Endophytic *Lecanicillium lecanii* and *Beauveria bassiana* reduce the survival and fecundity of *Aphis gossypii* following contact with conidia and secondary metabolites. Crop Prot.

[CR39] Gutierrez RMP, Gonzalez AMN, Ramirez AM (2012). Compounds derived from endophytes: a review of phytochemistry and pharmacology. Curr Med Chem.

[CR40] Gwynn RL (2014). The manual of biocontrol agents: a world compendium.

[CR41] Hallmann J, Sikora RA (1996). Toxicity of fungal endophyte secondary metabolites to plant parasitic nematodes and soil-borne plant pathogenic fungi. Eur J Plant Pathol.

[CR42] Hamdi F, Fargues J, Ridray G, Jeannequin B, Bonato O (2011). Compatibility among entomopathogenic hyphocreales and two beneficial insects used to control *Trialeurodes vaporariorum* (Hemiptera: Aleurodidae) in Mediterranean greenhouses. J Invertebr Pathol.

[CR43] Hartfield CM, Campbell CAM, Hardie J, Pickett JA, Wadhams LJ (2001). Pheromone traps for the dissemination of an entomopathogen by the Damson-hop aphid *Phorodon humuli* Biocontrol. Sci Technol.

[CR44] Heinz KM, Van Driesche RG, Parella MP (2004). Biocontrol in protected culture.

[CR45] Hemming S, Kempkes FLK, Janse J (2012) New greenhouse concept with high insulating double glass and new climate control strategies—Modelling and first results from a cucumber experiment. In: Acta Horticulturae. International Society for Horticultural Science (ISHS), Leuven, Belgium, pp 231–239

[CR46] Hendry TA, Hunter MS, Baltrusa DA (2014). The facultative symbiont *Rickettsia* protects an invasive whitefly against entomopathogenic *Pseudomonas syringae* Strains. Appl Environ Microbiol.

[CR47] Hibbett DS (2007). A higher-level phylogenetic classification of the Fungi. Mycol Res.

[CR48] Hosokawa T, Kikuchi Y, Shimada M, Fukatsu T (2007). Obligate symbiont involved in pest status of host insect. Proc R Soc B-Biol Sci.

[CR49] Huang NX, Enkegaard A, Osborne LS, Ramakers PMJ, Messelink GJ, Pijnakker J, Murphy G (2011). The banker plant method in biological control. Crit Rev Plant Sci.

[CR50] Humber RA (2012). Entomophthoromycota: a new phylum and reclassification for entomophthoroid fungi. Mycotaxon.

[CR51] Jakobsen L, Brogaard M, Enkegaard A, Brodsgaard HF (2006). Dynamic and traditional greenhouse climate regimes: Influx of thrips (Thysanoptera). HortScience.

[CR52] Jallow MFA, Dugassa-Gobena D, Vidal S (2004). Indirect interaction between an unspecialized endophytic fungus and a polyphagous moth. Basic Appl Ecol.

[CR53] Janssen A, Pallini A, Venzon M, Sabelis MW (1998). Behaviour and indirect interactions in food webs of plant-inhabiting arthropods. Exp Appl Acarol.

[CR54] Johansen NS, Vänninen I, Pinto DM, Nissinen AI, Shipp L (2011). In the light of new greenhouse technologies: 2. Direct effects of artificial lighting on arthropods and integrated pest management in greenhouse crops. Ann Appl Biol.

[CR55] Kapongo JP, Shipp L, Kevan P, Sutton JC (2008). Co-vectoring of *Beauveria bassiana* and *Clonostachys rosea* by bumble bees (*Bombus impatiens*) for control of insect pests and suppression of grey mould in greenhouse tomato and sweet pepper. Biol Control.

[CR56] Kaur T, Singh B, Kaur A, Kaur S (2015). Endophyte-mediated interactions between cauliflower, the herbivore *Spodoptera litura*, and the ectoparasitoid *Bracon hebetor*. Oecologia.

[CR57] Kavino M, Harish S, Kumar N, Saravanakumar D, Damodaran T, Soorianathasundaram K, Samiyappan R (2007). Rhizosphere and endophytic bacteria for induction of systemic resistance of banana plantlets against bunchy top virus. Soil Biol Biochem.

[CR58] Kaya HK, Gaugler R (1993). Entomopathogenic nematodes. Annu Rev Entomol.

[CR59] Kennedy GG (2003). Tomato, pests, parasitoids, and predators: Tritrophic interactions involving the genus Lycopersicon. Annu Rev Entomol.

[CR60] Keyser CA, Thorup-Kristensen K, Meyling NV (2014). Metarhizium seed treatment mediates fungal dispersal via roots and induces infections in insects. Fungal Ecol.

[CR61] Khan S, Guo L, Maimaiti Y, Mijit M, Qiu D (2012). Entomopathogenic fungi as microbial biocontrol agent. Mol Plant Breed.

[CR62] Kikuchi Y, Hosokawa T, Fukatsu T (2011). Specific developmental window for establishment of an insect-microbe gut symbiosis. Appl Environ Microbiol.

[CR63] Kim HY (2007). Some fungal endophytes from vegetable crops and their anti-oomycete activities against tomato late blight. Lett Appl Microbiol.

[CR64] Klein MG, Lacey LA (1999). An attractant trap for autodissemination of entomopathogenic fungi into populations of the Japanese beetle *Popillia japonica* (Coleoptera:Scarabaeidae). Biocontrol Sci Technol.

[CR65] Kloepper JW, Ryu C-M, Schulz BJE, Boyle CJC, Sieber TN (2006). Bacterial endophytes as elicitors of induced systemic resistance. Microbial root endophytes.

[CR66] Knight AL, Witzgall P (2013). Combining mutualistic yeast and pathogenic Virus—A novel method for codling moth control. J Chem Ecol.

[CR67] Körner O, Challa H (2003). Design for an improved temperature integration concept in greenhouse cultivation. Comput Electron Agric.

[CR68] Kreutz J, Zimmermann G, Vaupel O (2004). Horizontal transmission of the entomopathogenic fungus *Beauveria bassiana* among the spruce bark beetle, *Ips typographus* (Col., Scolytidae) in the laboratory and under field conditions. Biocontrol Sci Technol.

[CR69] Labbé RM, Gillespie DR, Cloutier C, Brodeur J (2009). Compatibility of an entomopathogenic fungus with a predator and a parasitoid in the biological control of greenhouse whitefly Biocontrol. Sci Technol.

[CR70] Lacey LA, Grzywacz D, Shapiro-Ilan DI, Frutos R, Brownbridge M, Goettel MS (2015). Insect pathogens as biological control agents: Back to the future. J Invertebr Pathol.

[CR71] Landis DA, Wratten SD, Gurr GM (2000). Habitat management to conserve natural enemies of arthropod pests in agriculture. Annu Rev Entomol.

[CR72] Lecuona RE, Turica M, Tarocco F, Crespo DC (2005). Microbial control of *Musca domestica* (Diptera:Muscidae) with selected strains of *Beauveria bassiana*. J Med Entomol.

[CR73] Lohse R, Jakobs-Schonwandt D, Vidal S, Patel AV (2015). Evaluation of new fermentation and formulation strategies for a high endophytic establishment of *Beauveria bassiana* in oilseed rape plants. Biol Control.

[CR74] Lopes RB, Laumann RA, Moore D, Oliveira MWM, Faria M (2014). Combination of the fungus *Beauveria bassiana* and pheromone in an attract-and-kill strategy against the banana weevil, *Cosmopolites sordidus*. Entomol Exp Appl.

[CR75] Ludwig SW, Oetting RD (2001). Susceptibility of natural enemies to infection by *Beauveria bassiana* and impact of insecticides on *Ipheseius degenerans* (Acari:Phytoseiidae). J Agric Urban Entomol.

[CR76] Łukasik P, van Asch M, Guo H, Ferrari J, Charles J (2013). Godfray H. Unrelated facultative endosymbionts protect aphids against a fungal pathogen Ecol Lett.

[CR77] Markkula M, Tiittanen K (1976). "Pest-in-First” and “natural infestation” methods in the control of *Tetranychus urticae* Koch with *Phytoseiulus persimilis* Athias-Henriot on glasshouse cucumbers. Ann Entomol Fenn.

[CR78] Markkula M, Tiittanen K, Hamalainen M, Forsberg A (1979). The aphid midge Aphidoletes aphidimyza (Diptera, Cecidomyiidae) and its use in biological control of aphids. Ann Entomol Fenn.

[CR79] Martinuz A, Schouten A, Menjivar RD, Sikora RA (2012). Effectiveness of systemic resistance toward *Aphis gossypii* (Hom., Aphididae) as induced by combined applications of the endophytes *Fusarium oxysporum* Fo162 and *Rhizobium etli* G12. Biol Control.

[CR80] Mesquita ALM, Lacey LA (2001). Interactions among the entomopathogenic fungus, *Paecilomyces fumosoroseus* (Deuteromycotina:Hyphomycetes), the parasitoid, *Aphelinus asychis* (Hymenoptera:Aphelinidae), and their aphid host. Biol Control.

[CR81] Messelink GJ, van Maanen R, van Steenpaal SEF, Janssen A (2008). Biological control of thrips and whiteflies by a shared predator: two pests are better than one. Biol Control.

[CR82] Messelink GJ, Van Maanen R, Van Holstein-Saj R, Sabelis MW, Janssen A (2010). Pest species diversity enhances control of spider mites and whiteflies by a generalist phytoseiid predator. Biocontrol.

[CR83] Messelink GJ, Sabelis MW, Janssen A, Larramendy ML, Soloneski S (2012). Generalist predators, food web complexities and biological pest control in greenhouse crops. Integrated pest management and pest control—current and future tactics.

[CR84] Messelink GJ (2014). Approaches to conserving natural enemy populations in greenhouse crops: current methods and future prospects. Biocontrol.

[CR85] Meyling NV, Pell JK (2006). Detection and avoidance of an entomopathogenic fungus by a generalist insect predator. Ecol Entomol.

[CR86] Mfuti DK (2016). Spatial separation of semiochemical Lurem-TR and entomopathogenic fungi to enhance their compatibility and infectivity in an autoinoculation system for thrips management. Pest Manag Sci.

[CR87] Milner RJ, Holdom DG, Glare TR (1984). Diurnal patterns of mortality in aphids infected by entomophthoran fungi. Entomol Exp Appl.

[CR88] Mohan MC, Reddy NP, Devi UK, Kongara R, Sharma HC (2007). Growth and insect assays of *Beauveria bassiana* with neem to test their compatibility and synergism Biocontrol. Sci Technol.

[CR89] Montero JI, Henten EJV, Son JE (2011). Henten EJv, Son JE, Castilla N. Greenhouse engineering: new technologies and approaches Acta Horticulturae.

[CR90] Montllor CB, Maxmen A, Purcell AH (2002). Facultative bacterial endosymbionts benefit pea aphids *Acyrthosiphon pisum* under heat stress. Ecol Entomol.

[CR91] Müller CB, Williams IS, Hardie J (2001). The role of nutrition, crowding and interspecific interactions in the development of winged aphids. Ecol Entomol.

[CR92] Muñoz-Cárdenas K, Fuentes LS, Cantor RF, Rodríguez CD, Janssen A, Sabelis MW (2014). Generalist red velvet mite predator (*Balaustium* sp.) performs better on a mixed diet. Exp Appl Acarol.

[CR93] Muvea AM, Meyhöfer R, Subramanian S, Poehling H-M, Ekesi E, Maniania NK (2014). Colonization of onions by endophytic fungi and their impacts on the biology of Thrips tabaci. PLOSone.

[CR94] Niassy S, Maniania NK, Subramanian S, Gitonga LM, Ekesi S (2012). Performance of a semiochemical-baited autoinoculation device treated with *Metarhizium anisopliae* for control of *Frankliniella occidentalis* on French bean in field cages. Entomol Exp Appl.

[CR95] Oliver KM, Russell JA, Moran NA, Hunter MS (2003). Facultative bacterial symbionts in aphids confer resistance to parasitic wasps. Proc Natl Acad Sci USA.

[CR96] Oliver KM, Smith AH, Russell JA (2014). Defensive symbiosis in the real world—advancing ecological studies of heritable, protective bacteria in aphids and beyond. Funct Ecol.

[CR97] Ownley BH, Griffin MR, Klingeman WE, Gwinn KD, Moulton JK, Pereira RM (2008). *Beauveria bassiana*: endophytic colonization and plant disease control. J Invertebr Pathol.

[CR98] Pell JK, Macaulay EDM, Wilding N (1993). A pheromone trap for dispersal of the pathogen *Zoophthora radicans* Brefeld. (Zygomycetes: Entomophthorales) amongst populations of the diamondback moth, *Plutella xylostella* L. (Lepidoptera: Yponomeutidae) Biocontrol. Sci Technol.

[CR99] Pell JK, Pluke R, Clark SJ, Kenward MG, Alderson PG (1997). Interactions between two aphid natural enemies, the entomopathogenic fungus *Erynia neoaphidis* Remaudiere & Hennebert (Zygomycetes: Entomophthorales) and the predatory beetle *Coccinella septempunctata* L (Coleoptera: Coccinellidae). J Invertebr Pathol.

[CR100] Pell JK, Eilenberg J, Hajek AE, Steinkraus DC, Butt TM, Jackson C, Magan N (2001). Biology, ecology and pest management potential of *Entomophthorales*. Fungi as biocontrol agents: progress, problems and potential.

[CR101] Pell JK, Hannam JJ, Steinkraus DC (2010). Conservation biological control using fungal entomopathogens. BioControl.

[CR102] Pickett JA, Wadhams LJ, Woodcock CM, Hardie J (1992). The chemical ecology of aphids. Annu Rev Entomol.

[CR103] Pilkington LJ, Messelink G, van Lenteren JC, Le Mottee K (2010). “Protected Biological Control”—Biological pest management in the greenhouse industry. Biol Control.

[CR104] Posada F, Aime MC, Peterson SW, Rehner SA, Vega FE (2007). Inoculation of coffee plants with the fungal entomopathogen *Beauveria bassiana* (Ascomycota: Hypocreales). Mycol Res.

[CR105] Pourian HR, Talaei-Hassanloui R, Kosari AA, Ashouri A (2011). Effects of *Metarhizium anisopliae* on searching, feeding and predation by *Orius albidipennis* (Hem., Anthocoridae) on *Thrips tabaci* (Thy., Thripidae) larvae. Biocontrol Sci Technol.

[CR106] Price PW, Bouton CE, Gross P, McPheron BA, Thompson JN, Weis AE (1980). interactions among three trophic levels: influence of plants on interactions between insect herbivores and natural enemies. Annu Rev Ecol Syst.

[CR107] Qayyum MA, Wakil W, Arif MJ, Sahi ST, Dunlap CA (2015). Infection of *Helicoverpa armigera* by endophytic *Beauveria bassiana* colonizing tomato plants. Biol Control.

[CR108] Rännbäck LM, Cotes B, Anderson P, Rämert B, Meyling NV (2015). Mortality risk from entomopathogenic fungi affects oviposition behavior in the parasitoid wasp Trybliographa rapae. J Invertebr Pathol.

[CR109] Raps A, Vidal S (1998). Indirect effects of an unspecialized endophytic fungus on specialized plant—herbivorous insect interactions. Oecologia.

[CR110] Ravensberg WJ (2011) A Roadmap to the successful development and commercialization of microbial pest control products for control of arthropods. Progress in Biological Control, 10. Springer Science + Business Media B.V., Dordrecht

[CR111] Reddy NP, Khan PAA, Devi KU, Victor JS, Sharma HC (2008). Assessment of the suitability of Tinopal as an enhancing adjuvant in formulations of the insect pathogenic fungus *Beauveria bassiana* (Bals.) Vuillemin. Pest Manag Sci.

[CR112] Roditakis E, Couzin ID, Franks NR, Charnley AK (2008). Effects of *Lecanicillium longisporum* infection on the behaviour of the green peach aphid *Myzus persicae*. J Insect Physiol.

[CR113] Rodriguez RJ, White JF, Arnold AE, Redman RS (2009). Fungal endophytes: diversity and functional roles. New Phytol.

[CR114] Roy HE, Pell JK (2000). Interactions between entomopathogenic fungi and other natural enemies: implications for biological control Biocontrol. Sci Technol.

[CR115] Roy HE, Pell JK, Alderson PG (2001). Targeted dispersal of the aphid pathogenic fungus *Erynia neoaphidis* by the aphid predator *Coccinella septempunctata* Biocontrol. Sci Technol.

[CR116] Roy HE, Steinkraus DC, Eilenberg J, Hajek AE, Pell JK (2006). Bizarre interactions and endgames: Entomopathogenic fungi and their arthropod hosts. Annu Rev Entomol.

[CR117] Roy HE, Baverstock J, Ware RL, Clark SJ, Majerus MEN, Baverstock KE, Pell JK (2008). Intraguild predation of the aphid pathogenic fungus *Pandora neoaphidis* by the invasive coccinellid *Harmonia axyridis*. Ecol Entomol.

[CR118] Ryan RP, Germaine K, Franks A, Ryan DJ, Dowling DN (2008). Bacterial endophytes: recent developments and applications. FEMS Microbiol Lett.

[CR119] Saito T, Brownbridge M (2016). Compatibility of soil-dwelling predators and microbial agents and their efficacy in controlling soil-dwelling stages of western flower thrips *Frankliniella occidentalis*. Biol Control.

[CR120] Sanahuja G, Banakar R, Twyman RM, Capell T, Christou P (2011). *Bacillus thuringiensis*: a century of research, development and commercial applications. Plant Biotechnol J.

[CR121] Schulz B, Haas S, Junker C, Andree N, Schobert M (2015). Fungal endophytes are involved in multiple balanced antagonisms. Curr Sci.

[CR122] Shah PA, Clark SJ, Pell J (2004). Assessment of aphid host range and isolate variability in *Pandora neoaphidis* (Zygomycetes: Entomophthorales). Biol Control.

[CR123] Shapiro M (1992). Use of optical brighteners as radiation protectants for gypsy moth (Lepidoptera, Lymantriidae) nuclear polyhedrosis virus. J Econ Entomol.

[CR124] Shipp JL, Zhang Y, Hunt DWA, Ferguson G (2003). Influence of humidity and greenhouse microclimate on the efficacy of *Beauveria bassiana* (Balsamo) for control of greenhouse arthropod pests. Environ Entomol.

[CR125] Shipp L, Kapongo JP, Park HH, Kevan P (2012). Effect of bee-vectored *Beauveria bassiana* on greenhouse beneficials under greenhouse cage conditions. Biol Control.

[CR126] Skinner M, Gouli S, Frank CE, Parker BL, Kim JS (2012). Management of *Frankliniella occidentalis* (Thysanoptera: Thripidae) with granular formulations of entomopathogenic fungi. Biol Control.

[CR127] Sturz AV, Christie BR, Nowak J (2000). Bacterial endophytes: potential role in developing sustainable systems of crop production. Crit Rev Plant Sci.

[CR128] Tefera T, Vidal S (2009). Effect of inoculation method and plant growth medium on endophytic colonization of sorghum by the entomopathogenic fungus *Beauveria bassiana*. Biocontrol.

[CR129] Teixeira L, Ferreira A, Ashburner M (2008). The bacterial symbiont wolbachia induces resistance to RNA viral infections in Drosophila melanogaster. Plos Biol.

[CR130] Tinzaara W, Gold CS, Dicke M, Van Huis A, Nankinga CM, Kagezi GH, Ragama PE (2007). The use of aggregation pheromone to enhance dissemination of *Beauveria bassiana* for the control of the banana weevil in Uganda. Biocontrol Sci Technol.

[CR131] Tsutsumi T, Teshiba M, Yamanaka M, Ohira Y, Higuchi T (2003). An autodissemination system for the control of brown winged green bug, *Plautia crossota**stali* Scott (Heteroptera: Pentatomidae) by an entomopathogenic fungus, *Beauveria bassiana* E-9102 combined with aggregation pheromone. Jpn J Appl Entomol Zool.

[CR132] Vachon V, Laprade R, Schwartz JL (2012). Current models of the mode of action of *Bacillus thuringiensis* insecticidal crystal proteins: a critical review. J Invertebr Pathol.

[CR133] Vadiee A, Martin V (2014). Energy management strategies for commercial greenhouses. Appl Energy.

[CR134] Valenzuela-Soto JH, Estrada-Hernandez MG, Ibarra-Laclette E, Delano-Frier JP (2010). Inoculation of tomato plants (*Solanum lycopersicum*) with growth-promoting *Bacillus subtilis* retards whitefly *Bemisia tabaci* development. Planta.

[CR135] Van den Heuvel JFJM, Verbeek M, Vanderwilk F (1994). Endo-symbiotic bacteria associated with circulative transmission of potato leafroll virus by *Myzus persicae*. J Gen Virol.

[CR136] Van der Putten WH, Vet LE, Harvey JA, Wäckers FL (2001). Linking above-and belowground multitrophic interactions of plants, herbivores, pathogens, and their antagonists. Trends Ecol Evol.

[CR137] van Henten EJ (2006). The adaptive greenhouse—an integrated systems approach to developing protected cultivation systems. Acta Horticult.

[CR138] van Lenteren JC (2012). The state of commercial augmentative biological control: plenty of natural enemies, but a frustrating lack of uptake. Biocontrol.

[CR139] Vangansbeke D, Audenaert J, Nguyen DT, Verhoeven R, Gobin B, Tirry L, De Clercq P (2015) Diurnal temperature variations affect development of a herbivorous arthropod pest and its predators. PLoS One 10 10.1371/journal.pone.0124898 PMC4398551 25874697

[CR140] Vega FE, Dowd PF, Bartelt RJ (1995). Dissemination of microbial agents using an autoinoculating device and several insect species as vectors. Biol Control.

[CR141] Vega FE, Dowd PF, Lacey LA, Pell JK, Jackson DM, Klein MG, Lacey LA, Kaya HK (2007). Dissemination of beneficial microbial agents by insects. Field manual of techniques in invertebrate pathology.

[CR142] Vega FE, Posada F, Aime MC, Pava-Ripoll M, Infante F, Rehner SA (2008). Entomopathogenic fungal endophytes. Biol Control.

[CR143] Vega FE (2009). Fungal entomopathogens: new insights on their ecology. Fungal Ecol.

[CR144] Vemmer M, Patel AV (2013). Review of encapsulation methods suitable for microbial biological control agents. Biol Control.

[CR145] Vergel SJN, Bustos RA, Rodriguez CD, Cantor RF (2011). Laboratory and greenhouse evaluation of the entomopathogenic fungi and garlic-pepper extract on the predatory mites, *Phytoseiulus persimilis* and *Neoseiulus californicus* and their effect on the spider mite *Tetranychus urticae*. Biol Control.

[CR146] Vet LEM, Dicke M (1992). Ecology of infochemical use by natural enemies in a tritrophic context. Annu Rev Entomol.

[CR147] Vickers RA, Furlong MJ, White A, Pell JK (2004). Initiation of fungal epizootics in diamondback moth populations within a large field cage: proof of concept for auto-dissemination. Entomol Exp Appl.

[CR148] Vidal S (1996). Changes in suitability of tomato for whiteflies mediated by a non-pathogenic endophytic fungus. Entomol Exp Appl.

[CR149] Vidal S, Jaber LR (2015). Entomopathogenic fungi as endophytes: plant-endophyte-herbivore interactions and prospects for use in biological control. Curr Sci.

[CR150] Wells PM, Baverstock J, Majerus MEN, Jiggins FM, Roy HE, Pell JK (2011). The effect of the coccinellid *Harmonia axyridis* (Coleoptera: Coccinellidae) on transmission of the fungal pathogen *Pandora neoaphidis* (Entomophthorales: Entomophthoraceae) Eur. J Entomol.

[CR151] Werren JH, Baldo L, Clark ME (2008). *Wolbachia*: master manipulators of invertebrate biology. Nat Rev Microbiol.

[CR152] Wilson D (1995). Endophyte: the evolution of a term, and clarification of its use and definition. Oikos.

[CR153] Wu SY, Gao YL, Zhang YP, Wang ED, Xu XN, Lei ZR (2014). An entomopathogenic strain of *Beauveria bassiana* against *Frankliniella occidentalis* with no detrimental effect on the predatory mite *Neoseiulus barkeri*: evidence from laboratory Bioassay and scanning electron microscopic observation. PLoS One.

[CR154] Renn N, Bywater AF, Barson G (1999). A bait formulated with *Metarhizium anisopliae* for the control of *Musca domestica* L-(Dipt., Muscidae) assessed in large-scale laboratory enclosures. J Appl Entomol-Z Angew Entomol.

[CR155] Yan JF, Broughton SJ, Yang SL, Gange AC (2015). Do endophytic fungi grow through their hosts systemically?. Fungal Ecol.

[CR156] Yao SL, Ying SH, Feng MG, Hatting JL (2010). In vitro and in vivo responses of fungal biocontrol agents to gradient doses of UV-B and UV-A irradiation. Biocontrol.

[CR157] Yasuda K (1999). Auto-infection system for the sweet potato weevil, *Cylas formicarius* (Fabricius) (Coleoptera: Curculionidae) with entomopathogenic fungi, *Beauveria bassiana* using a modified sex pheromone trap in the field. Appl Entomol Zool.

[CR158] Zchori-Fein E, Bourtzis K (2012). Manipulative tenants: bacteria associated with arthropods. Frontiers in microbiology.

[CR159] Zhang Y-X et al. (2015) A novel use of predatory mites for dissemination of fungal pathogen for insect biocontrol: The case of *Amblyseius swirskii* and *Neoseiulus cucumeris* (Phytoseiidae) as vectors of *Beauveria bassiana* against *Diaphorina citri* (Psyllidae). Syst Appl Acarol 20

[CR160] Zindel R, Gottlieb Y, Aebi A (2011). Arthropod symbioses: a neglected parameter in pest- and disease-control programmes. J Appl Ecol.

